# Case report: A rare multidrug-resistant *Escherichia coli* causes fatal neonatal meningoencephalitis

**DOI:** 10.3389/fpubh.2023.1174536

**Published:** 2023-07-28

**Authors:** Qingyun Zhou, Zhifei Zhan, Furong Huang, Menghua Zhao, Daosheng Huang, Jun Xu, Li Huang, Liangyi Xie, Aimin Zhang

**Affiliations:** ^1^The First-Affiliated Hospital of Hunan Normal University, Hunan Provincial People's Hospital, Changsha, Hunan, China; ^2^Hunan Provincial Center for Disease Control and Prevention, Changsha, China

**Keywords:** encephalopathy, metagenomic next-generation sequencing (mNGS), infant, infectious disease, neonatal meningitis, *Escherichia coli*

## Abstract

Neonatal meningitis is rare but devastating disease. Multidrug-resistant (MDR, multi-drug resistant) bacteria are a major global health risk. We report an *Escherichia coli* meningitis isolate with multiple resistance patterns and unusual serotype (O75) that caused sudden neonatal death. The isolate was resistant to antibiotics other than cefoperazone/sulbactam and imipenem, challenging the combination of antibiotics commonly used in the empirical treatment of neonatal sepsis. Despite aggressive symptomatic and supportive treatment of the infant based on laboratory tests and clinical practice, the infant eventually died. This is the first case of meningoencephalitis due to serotype O75 reported in China. The presence of highly pathogenic multidrug-resistant microorganisms isolated in neonates underscores the need to implement rapid resistance diagnostic methods and should prompt consideration of alternatives to empiric treatment of neonatal bacterial meningitis.

## Introduction

Neonatal meningoencephalitis is an inflammatory disease that seriously endangers the lives of newborns (1). Bacterial neonatal meningoencephalitis causes acute inflammation of the neonatal brain tissue, cerebrospinal membrane, subarachnoid space and cerebrovascular system. Cases of neonatal refractory bacterial meningoencephalitis (BM) are dominated by *Escherichia coli* and B *Streptococci* infections, with *E. coli* being the leading cause of death (2–5). The main reason for this is the multi-drug resistance (MDR) of *E. coli*. While accordingly, information on the bacterial factors of unique serotype required for the treatment is limited, and most of the associated virulence factors have not been described (6).

This study reports a case of invasive lethal neonatal meningitis caused by MDR *E. coli* (serotype O75). The strain is highly virulent, resistant to ampicillin and gentamicin, third-generation cephalosporins, and susceptible only to meropenem and cefoperazone sulbactam. This is the second time globally and the first report of meningitis due to *E. coli* O75 infection in China. After aggressive treatment, the disease progressed drastically and died due to ineffective treatment. Metagenomic next-generation sequencing (mNGS) as an unbiased and hypothesis-free pathogen detection method was beneficial in this case for identifying pathogens and detecting resistance and resistance genes (7).

## Case description

On January 19th, 2022, a 19-day-old baby boy was admitted to the Pediatric Intensive Care Unit of Hunan Provincial People's Hospital because of intermittent crying for 3 days, convulsions for 23 h and fever for 18 h.

The infant is the first-born child and was delivered vaginally at 38 weeks on December 31th, 2021, with a birth weight of 2.52 kg. No examination or research has been conducted on the maternal bacterial colonization status. Three days ago, the infant cried intermittently for no apparent reason, could be soothed, had no fever, vomiting, cough or other discomfort, and had not been treated specifically. In the early morning of January 19th, he presented with fever (temperature 38.6°C), convulsions, trembling limbs, dull gaze, no cyanosis, foaming at the mouth, occasional spitting up, no choking cough, low milk intake, and stools 4–5 times/day. He was immediately admitted to the local hospital, given “cefotaxime, penicillin, phenobarbital to stop fright and furosemide to diuretic,” and transferred to our hospital.

**Figure 1:**
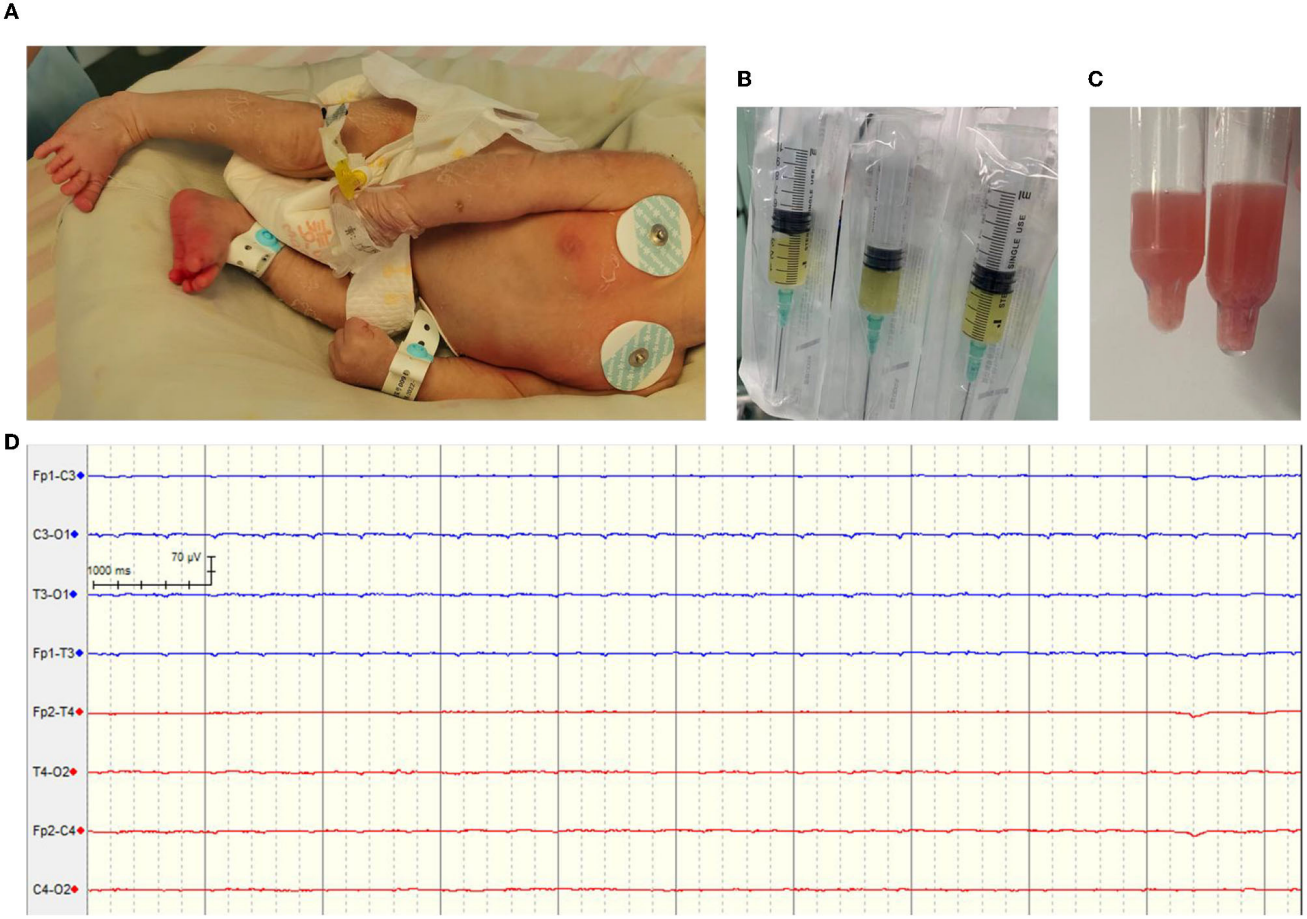
**(A)** The infant presents with convulsions and corneal regurgitation. **(B)** Cerebrospinal fluid sample on the day of admission. **(C)** Drained cerebrospinal fluid sample. **(D)** Electroencephalogram of the infant on the 10th day of admission.

**Figure 2:**
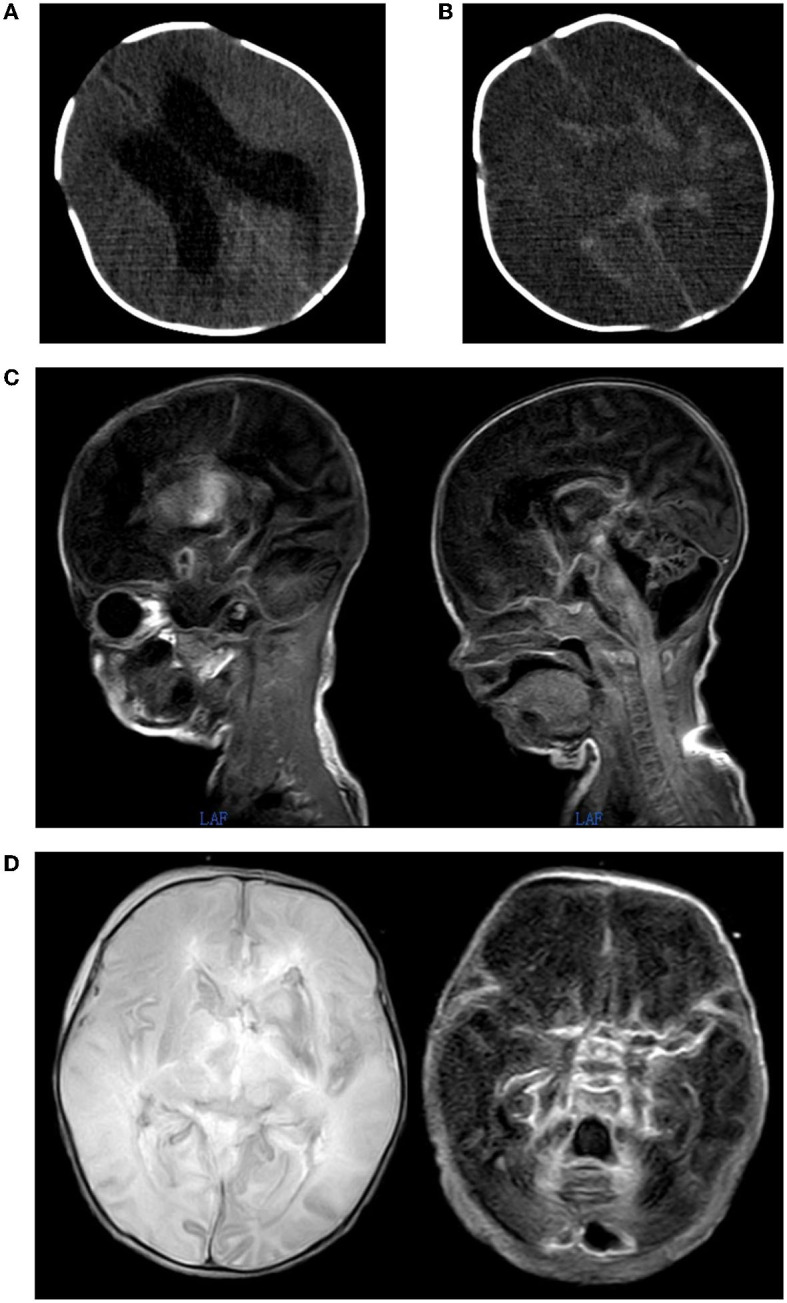
**(A)** On 2022.01.19th, head CT shows no obvious abnormal density lesions in the brain parenchyma, enlargement of the ventricular with lateral ventricular dilatation as the main feature, no displacement of midline structures and no widening of the sulcus pool. **(B)** On 2022.01.24th, head CT scan showed severe cerebral edema and significant shrinkage of the ventricular compared with before. **(C, D)** On 2022.01.26th, MRI scan of the head showed changes in the left ventricle after puncture drainage. Severe cerebral edema suggested the presence of ventricular and subarachnoid hemorrhage.

**Figure 3:**
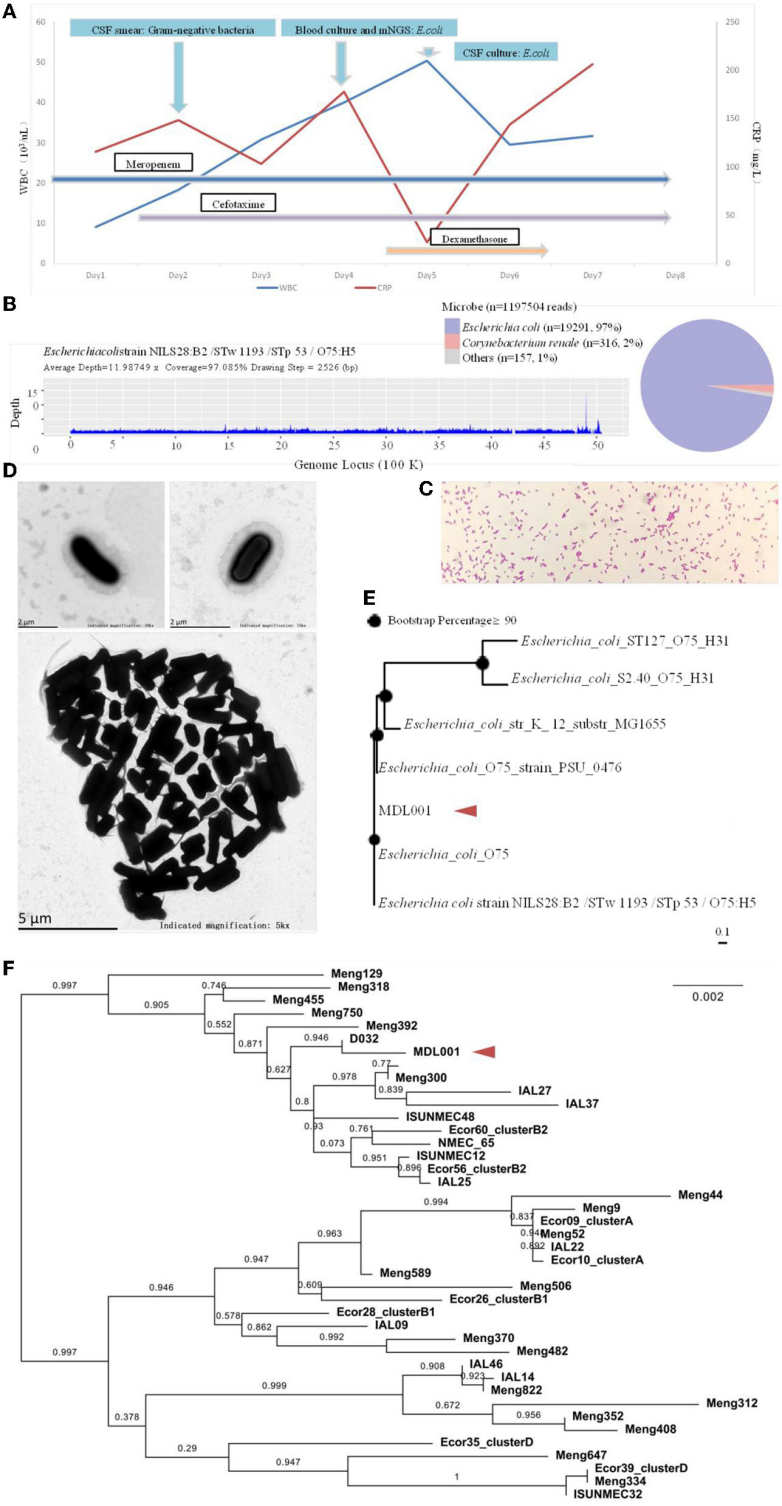
**(A)** Schematic diagram of the patient's inflammatory factors, pathogen detection results and medication administration over time. **(B)** On day 4 of admission, the cerebrospinal fluid mNGS showed 19291 specific sequences with 97% coverage. **(C, D)** Light and electron microscopic observations of the pure culture of the pathogen. **(E)** The genomic -based homology analysis of the pathogen based on mNGS sequences. **(F)** The MLST homology analysis of the pathogen based on mNGS sequences.

On admission, the infant had a body temperature of 39.7 °C, angular arches (Figure 1A). The muscle tone of the limbs was high and the physiological reflexes disappeared. The anterior fontanelle was 2.5 × 2.5 cm, elevated, with high tension. The pupils were 3 mm bilaterally, equilaterally round, with a blunted reflex to light. There was no sick person in the family and no history of suspicious patient contact. A CT head examination was performed (Figure 2A), which showed no significant abnormal density lesions in the patient's brain parenchyma, thus intracranial hemorrhage and transient lesions were ruled out and a preliminary diagnosis of hydrocephalus was made. He was immediately treated with meropenem combined with cefsevox, mannitol to lower cranial pressure, and nutritional support (Figure 3A). Due to frequent convulsions, he was given imipramine and etiracetam as anticonvulsants and supported by mechanical ventilation.

On day 2 of admission, the infant was in a comatose state with a left pupil of approximately 4 mm and a right pupil of approximately 1 mm, and the reflex to light was absent. Cerebrospinal fluid (CSF) examination revealed a yellowish, cloudy color (Figure 1B), and a smear showed Gram-negative bacteria infection. The Pandy test result was 1+ (increased), the white blood cell count was 1.2 × 10^9^/L (increased), the red blood cell count was 1.7 × 10^9^/L (increased), the neutrophil count was 0.7 × 10^9^/L, and the lymphocyte count was 0.3 × 10^9^/L. Additionally, the patient's glucose level was noted to be 0.01 mmol/L (decreased), with normal levels of chlorine (98.00 mmol/L) and elevated levels of lactate dehydrogenase (956.45 U/L). Finally, cerebrospinal fluid or urine protein was found to be 2,090.40 mg/L. The fontanelle was full and tense. The possibility of brain herniation was considered, and emergency lateral ventriculotomy drainage was performed. The drainage fluid turned light red with flocculent material on January 21st (Figure 1C), but the fever was repeated, the limbs were tonic and shaking.

On day 3 of admission, blood culture detected *E. coli* (Figure 3A). The isolate was initially identified by Vitek 2 and further confirmed using MALDI-TOF MS (Vitek MS, BioMe'rieux, France) in accordance with the Clinical and Laboratory Standards Institute (CLSI) standard M100-S29 (CLSI, 2021). The next day, *E. coli* was also detected in CSF culture and mNGS (Hangzhou Matridx Biotechnology Co.,Ltd.; Figure 3B). The latter was carried out on illumina Nextseq. For each sequencing run, a negative control (culture medium containing 104 Jurkat cells/mL) was included. Microbial reads identified from a library were reported if: 1) the sequencing data passed quality control filters (library concentration > 50 pM, Q20 > 85%, Q30 > 80%); 2) negative control (NC) in the same sequencing run does not contain the species or the RPM (sample)/RPM (NC) ≥ 5 as a cutoff for discriminating true-positives from background contaminations The mNGS also detected multiple virulence genes and drug resistance genes.

A CT scan of the head on day 6 (Figure 2B) and an MRI scan of the head on day 8 (Figures 2C, D) showed that the ventricular was significantly smaller, and the lateral ventricular drainage tube was removed on day 8. From the ninth day of admission, the blood pressure dropped repeatedly with blood oxygenation and decreased heart rhythm, dobutamine epinephrine was given with continuous pumping and volume expansion, and the video electroencephalogram showed no significant electroencephalogram activity (Figure 1D). On day 11 of admission, the infant was in a deep coma. Infection indicators gradually increased, the condition was irreversible, and treatment was abandoned.

Subsequently, exploration of pure cultures of the pathogen further confirmed that the Gram-negative bacterium exhibited podocytes in electron microscopic observations (Figures 3C, D). The Hunan Provincial Center for Disease Control confirmed that the serotype of the pathogen is O75 using the serum agglutination method. This result was also confirmed by genomic homology analysis of the sequence obtained by sequencing in the mNGS results (Figures 3E, F). This serotype is very rare and has a low incidence, but is very virulent and highly resistant to drugs. Phenotypic characteristics and drug susceptibility testing confirmed its MDR (Tables 1, 2), while a large number of virulence and resistance genes were annotated in its genome sequence (Table 3).

**Table 1:** Phenotypic characteristics of the pathogen.

**Features**	**Detection**
Serotype	O75
Gram staining	Negative
Capsule	Positive
Biofilm	Negative

**Table 2:** Results of drug sensitivity tests for the pathogen.

**Drug sensitivity**	**Sensitivity**	**MIC**
Ticarcillin/Clavulanic acid	I	32
Aminotrans	S	4
Meropenem	S	<0.25
Tobramycin	I	8
Ciprofloxacin	R	>4.0
Doxycycline	S	<0.5
Mucomycin (polymyxin B)	S	≤ 0.5
Minocycline	R	>1.0
ESBL	+	
Amoxicillin Clavulanate Potassium	S	8
Piperacillin sulbactam	S	<4.0
Cefuroxime	R	>64
Cefuroxime	R	>64
Cefoxitin	S	<4.0
Ceftazidime	S	0.5
Ceftriaxone	R	>64
Cefoperazone sodium sulbactam sodium	S	16
Cefepime	R	>64
Ertapenem	S	<0.12
Imipenem	S	<0.25
Amikacin	S	<0.2
Levofloxacin	R	>8.0
Tigecycline	S	<0.5
Cotrimoxazole	S	<20

MIC, Minimum inhibition concentration. The results were interpreted according to Clinical and Laboratory Standards Institute (CLSI) standard M100-S29 (CLSI, 2021). Isolates were first identified and Antimicrobial Susceptibility Test by Vitek 2 Compact, further identification using MALDI-TOF MS (Vitek MS, BioMe ìrieux, France).

**Table 3:** Resistance genes and virulence factor genes of the pathogen detected by mNGS.

**Gene name**	**Reads number**	**Drug resistance categories**
**Resistance gene**		
*AAC(3)-IId*	217	Aminoglycoside antibiotic
*CRP*	98	Fluoroquinolone antibiotic; macrolide antibiotic; penam
*Escherichia coli acrA*	219	Cephalosporin; cephamycin; penam
*Escherichia coli ampC*	186	Cephalosporin; fluoroquinolone antibiotic; glycylcycline; penam; phenicol antibiotic; rifamycin antibiotic; tetracycline antibiotic; triclosan
*Escherichia coli ampH*	161	Cephalosporin; penam
*Escherichia coli emrE*	20	Cephalosporin; penam
*Escherichia coli mdfA*	126	Macrolide antibiotic
*H-NS*	112	Benzalkonium chloride; rhodamine; tetracycline antibiotic
*acrB*	506	Cephalosporin; cephamycin; fluoroquinolone antibiotic; macrolide antibiotic; penam; tetracycline antibiotic
*acrD*	417	Carbapenem; cephalosporin; cephamycin; monobactam; penam; penem
*acrE*	218	Cephalosporin; fluoroquinolone antibiotic; glycylcycline; penam; phenicol antibiotic; rifamycin antibiotic; tetracycline antibiotic; triclosan
*acrF*	452	Aminoglycoside antibiotic
*acrS*	193	Cephalosporin; cephamycin; fluoroquinolone antibiotic; penam
*bacA*	170	Cephalosporin; cephamycin; fluoroquinolone antibiotic; penam
*baeR*	63	Cephalosporin; cephamycin; fluoroquinolone antibiotic; glycylcycline; penam; phenicol antibiotic; rifamycin antibiotic; tetracycline antibiotic; triclosan
*baeS*	33	Peptide antibiotic
*cpxA*	252	Aminocoumarin antibiotic; aminoglycoside antibiotic
*emrA*	165	Aminocoumarin antibiotic; aminoglycoside antibiotic
*emrB*	175	Aminocoumarin antibiotic; aminoglycoside antibiotic
*emrK*	223	Fluoroquinolone antibiotic
*emrR*	130	Fluoroquinolone antibiotic
*emrY*	321	Tetracycline antibiotic
*eptA*	138	Fluoroquinolone antibiotic
*evgA*	219	Tetracycline antibiotic
*evgS*	1,249	Peptide antibiotic
*gadW*	316	Fluoroquinolone antibiotic; macrolide antibiotic; penam; tetracycline antibiotic
*gadX*	124	Fluoroquinolone antibiotic; macrolide antibiotic; penam; tetracycline antibiotic
*kdpE*	45	Fluoroquinolone antibiotic; macrolide antibiotic; penam
*marA*	59	Fluoroquinolone antibiotic; macrolide antibiotic; penam
*mdtA*	63	Aminoglycoside antibiotic
*mdtB*	197	Carbapenem; cephalosporin; cephamycin; fluoroquinolone antibiotic; glycylcycline; monobactam; penam; penem; phenicol antibiotic; rifamycin antibiotic; tetracycline antibiotic; triclosan
*mdtC*	145	Aminocoumarin antibiotic
*mdtE*	190	Aminocoumarin antibiotic
*mdtF*	409	Aminocoumarin antibiotic
*mdtG*	151	Fluoroquinolone antibiotic; macrolide antibiotic; penam
*mdtH*	126	Fluoroquinolone antibiotic; macrolide antibiotic; penam
*mdtN*	49	Fosfomycin
*mdtO*	223	Fluoroquinolone antibiotic
*mdtP*	211	Acridine dye; disinfecting agents and intercalating dyes; nucleoside antibiotic
*msbA*	273	Acridine dye; disinfecting agents and intercalating dyes; nucleoside antibiotic
*pmrF*	111	Acridine dye; disinfecting agents and intercalating dyes; nucleoside antibiotic
*tolC*	348	Nitroimidazole antibiotic
*ugd*	221	Peptide antibiotic
*yojI*	196	Aminocoumarin antibiotic; aminoglycoside antibiotic; carbapenem; cephalosporin; cephamycin; fluoroquinolone antibiotic; glycylcycline; macrolide antibiotic; penam; penem; peptide antibiotic; phenicol antibiotic; rifamycin antibiotic; tetracycline antibiotic; triclosan
**Virulence factor gene**		
*fimB*	166	Adherence
*fimE*	169	Adherence
*fimA*	41	Adherence
*fimI*	122	Adherence
*fimC*	212	Adherence
*fimD*	552	Adherence
*fimF*	71	Adherence
*fimG*	69	Adherence
*fimH*	116	Adherence
*papB*	69	Adherence
*papI*	29	Adherence
*papA*	5	Adherence
*sat*	1,480	Effector delivery system
*chuS*	270	Nutritional/metabolic factor
*chuA*	496	Nutritional/metabolic factor
*chuT*	212	Nutritional/metabolic factor
*chuW*	228	Nutritional/metabolic factor
*chuX*	132	Nutritional/metabolic factor
*chuY*	105	Nutritional/metabolic factor
*chuU*	196	Nutritional/metabolic factor
*fepA*	351	Nutritional/Metabolic factor
*fepB*	153	Nutritional/metabolic factor
*fepC*	131	Nutritional/metabolic factor
*fepD*	145	Nutritional/metabolic factor
*fepE*	175	Nutritional/metabolic factor
*fepG*	97	Nutritional/metabolic factor
*entD*	35	Nutritional/metabolic factor
*entF*	527	Nutritional/metabolic factor
*entC*	195	Nutritional/metabolic factor
*entE*	265	Nutritional/metabolic factor
*entB*	149	Nutritional/metabolic factor
*entA*	92	Nutritional/metabolic factor
*iucD*	38	Nutritional/metabolic factor
*iucC*	202	Nutritional/metabolic factor
*ompA*	209	Invasion
*aslA*	245	Invasion
*papX*	158	Adherence
*yagY/ecpB*	78	Adherence
*ykgK/ecpR*	202	Adherence
*yagZ/ecpA*	65	Adherence
*yagX/ecpC*	224	Adherence
*yagW/ecpD*	308	Adherence
*yagV/ecpE*	120	Adherence
*sfaC*	31	Adherence
*chuS*	21	Nutritional/metabolic factor
*chuW*	24	Nutritional/metabolic factor
*chuY*	9	Nutritional/metabolic factor
*ibeB*	306	Invasion
*ibeC*	446	Invasion
*espL1*	97	Effector delivery system
*sfaX*	22	Adherence
*fes*	218	Nutritional/metabolic factor
*entS*	156	Nutritional/metabolic factor
*chuV*	222	Nutritional/metabolic factor
*fdeC*	762	Adherence
*cgsG*	106	Adherence
*cgsF*	97	Adherence
*cgsE*	98	Adherence
*cgsD*	176	Adherence
*csgB*	142	Adherence
*csgA*	69	Adherence
*csgC*	70	Adherence
*kpsF*	243	Invasion
*kpsE*	223	Invasion
*kpsD*	314	Invasion
*kpsU*	78	Invasion
*kpsC*	300	Invasion
*kpsS*	253	Invasion
*neuE*	893	Invasion
*neuC*	636	Invasion
*neuA*	762	Invasion
*neuB*	578	Invasion
*neuD*	319	Invasion
*kpsT*	358	Invasion
*kpsM*	246	Invasion

## Discussion

Although neonatal meningitis *E. coli* serotypes O7 and O18 have been widely reported (8)^1^, in-depth clinical information on serotype O75 strains is not readily available. This serotype is very rare and has a very low incidence, but is very virulent and highly resistant to drugs. A case of fatal neonatal meningitis caused by serotype O75 pathogen was first reported in 2018^2^ and was very similar to the present case. This case is the second reported case of meningitis caused by *E. coli* infection type O75 worldwide, and the infant died after treatment failed. However, although the *E. coli* strains that cause neonatal meningitis have a high genetic diversity high, their respective pathogenesis is only partially understood.^3^ This is an urgent issue to be addressed. Because a prospective study including 325 samples has pointed out that the molecular characteristics of rare and highly virulent strains are one of the risk factors for severe disease or death.^4^ Considering the low morbidity, high pathogenicity and high clinical mortality of the serotype O75, the currently insufficient clinical experience in the treatment of this serotype should be further improved (6).

According to the guidelines recommended by joint initiative of the European Centre for Disease Prevention and Control and the Centers for Disease Control and Prevention (9), the pathogen showing non-susceptibility to at least one agent in three or more antimicrobial categories were identified as MDR. Although the Committee on Infectious Diseases recommended the use of third-generation cephalosporins in cases of Gram-negative meningitis (10), a growing number of reports describe the widespread spread of MDR *E. coli* isolates that have breached this line of defense in neonatal intensive care units worldwide (11). The reason is that *E. coli* can produce plasmid-mediated Extended Spectrum Beta-Lactamases (ESBLs) and cephalosporinases (AmpC enzymes). ESBLs, as derivatives of bacterial serine proteases, can hydrolyze the β-lactam ring of antimicrobial drugs, leading to their ability to be resistant to β-lactam antibiotics and 3rd generation cephalosporins. The isolation rate of third-generation cephalosporin-resistant *E. coli* has reached 51. 6% so far (12), so it has been proposed that carbapenem antibiotics, plus enzyme-based combination antibiotics (cefoperazone/sulbactam, amoxicillin/stick acid) are needed to achieve the desired effect (13).

*E. coli* frequently causes BM (14). Epidemiological studies suggest that *E. coli* meningitis may be a complication of *E. coli* bacteremia in the neonatal period (2, 15), and in clinical practice, blood cultures are often used as a test for *E. coli* meningitis, with a sensitivity of 62% to 76.1% (16, 17). This may also be the situation in this case, as both blood and CSF cultures detected *E. coli*.

Studies have shown that decreased glucose levels in the CSF suggest poor prognosis in BM, and some studies have shown that CSF leukocytes >500 × 10^6^/L, CSF protein concentration >1.0 g/L, and CSF glucose levels <1.5 mmol/L are independent risk factors for poor prognosis in BM (18). The presence of coma or mild coma, convulsions, hypotension, white blood cell count <5 × 10^9^/L, absolute neutrophil value <1 × 10^9^/L, CSF glucose concentration <1.1 mmol/L or protein >3 g/L at the beginning of the illness are the main risk factors for death in neonatal BM (19). Romain Basmaci studied 325 cases of BM and found severe CSF hypoglycemia (CSF/glucose ratio <0.10) to be an independent risk factor for death (16). In our case, the child presented with decreased crying, rapid onset of febrile convulsions, coma, increased fontanelle tone, corneal posture, and decreased CSF glucose starting 16 days after birth, which requires attention in clinical practice.

Generally, cephalosporin combined with ampicillin or cefoperazone combined with sulbactam is used for neonatal meningitis. In cases where Gram-negative bacteria are resistant, third generation cephalosporins such as ceftazidime can be considered. For severe infections or if the pathogen is an ESBL producing organism, meropenem and ceftazidime can be used in combination. The 19-day-old newborn was admitted to the hospital based on significant and severe clinical symptoms. The diagnosis was confirmed as purulent meningitis with a poor prognosis. Therefore, the patient received treatment with meropenem and ceftazidime after admission to the hospital. Meropenem was administered at 20–40 mg/kg every 8 h, and ceftazidime at 75 mg/kg every 8 h. Initially, the patient was treated with cefoperazone and penicillin at a dose of 50 mg/kg every 8 h at the local hospital. However, for severe patients, the dose needs to be >200 mg/kg/day.

mNGS allows comprehensive and unbiased detection of all pathogenic microorganisms in clinical samples (7), and this case is an example of the successful application of this method by providing early and accurate detection of the pathogen. Genotype-based methods are expected to rapidly and accurately detect or confirm antimicrobial resistance. In line with this, the results of mNGS indicate the presence of the multiple resistance genes and drug resistant genes. Unfortunately, however, this kind of method can only detect resistance and not susceptibility. Therefore, phenotypic approaches in clinical practice will also continue to guide antibiotic therapy, although turnaround times remain long (20, 21).

In conclusion, we report an *E. coli* isolate from a neonate who died suddenly with a multidrug resistance pattern and an unusual serotype of BM. The isolate was resistant to antibiotics other than cefoperazone/sulbactam and imipenem and may be resistant to combinations of antibiotics commonly used in the empirical treatment of neonatal sepsis. We believe that this *E. coli* clinical isolate represents the tip of the iceberg of multi-drug resistance and/or neonatal sepsis caused by highly pathogenic Gram-negative bacteria and should be taken seriously.

## Data availability statement

The datasets presented in this study can be found in online repositories. The names of the repository/repositories and accession number(s) can be found below: https://www.ncbi.nlm.nih.gov/, accession number PRJNA979834.

## Ethics statement

The studies involving human participants were reviewed and approved by the Ethics Committee of Hunan Provincial people's Hospital, China. Written informed consent was obtained from the minor's legal guardian for the publication of any potentially identifiable images or data included in this article. Written informed consent to participate in this study was provided by the participants' legal guardian/next of kin.

## Author contributions

QZ and ZZ designed the paper. AZ and LX drafted the manuscript. FH, MZ, and DH analyzed the data. JX and LH revised the manuscript. All authors read and approved the final manuscript.

## Footnotes

^1^ https://www.ncbi.nlm.nih.gov/pmc/articles/pmid/25164788/pdf/?tool=EBI

^2^ https://www.ncbi.nlm.nih.gov/pmc/articles/pmid/25164788/?tool=EBI

^3^ https://europepmc.org/articles/PMC4155114

^4^ https://europepmc.org/articles/PMC4155114?pdf=render
